# Microscopic Observations on Form and Structure of the Worm *Enchytraeus buchholzi* (Clitellata: Enchytraeidae)

**DOI:** 10.1186/s40850-022-00123-y

**Published:** 2022-06-06

**Authors:** Limin Zhao, Xiuchao Xie, Dejing Chen, Guilan Ma, Ya Sun

**Affiliations:** Shaanxi University of Technology, College of Bioscience and Engineering / Shaanxi Key-Laboratory of Bioresources, #1, East 1st Ring Road, Hanzhong, 723001 Shaanxi China

**Keywords:** Digestive organ, DNA barcoding, *Enchytraeus buchholzi*, Genital organ, Microphotograph, Nervous system

## Abstract

**Background:**

The worm *Enchytraeus buchholzi* is a new record species for Shaanxi, China, and a key pest on American ginseng *Panax quinquefolium*. To distinguish the species, the authors prepared its whole mounts and paraffin-embedded sections, and microscopically observed, photographed and measured. Besides, we conducted an experimental study on its DNA barcode.

**Results:**

Cells, tissues and organs related to nervous, digestive, circulatory, excretory and reproductive systems were found, photomicrographed and described, including: prostomium, peristomium, segments, clitellum, pygidium, lateral and ventral chaetae; brain, cranial nerves, sensory papillae, ventral nerve cord; pharyngeal pad and glands, retractor muscles and muscular bundles, peptonephridia, esophagus, intestine; dorsal, lateral, ventral and intestinal parietal vessels, coelomocytes, coelomic cavity; nephridia, chloragogen cells; ovaries, groups of germ cells with developing oocytes, mature eggs, spermathecae; testes, seminal vesicles, sperm funnels, penial bulbs. Their shapes and sizes were given, and functions discussed briefly. The visual effect of staining specimens with hematoxylin plus eosin ranked the first, and that with acetocarmine the second.

**Conclusions:**

The supplementary and objective descriptions, with the microphotographs as forceful pieces of evidence, have expanded biological knowledge in aspects of the form, structure and function of the worm, which is helpful for professionals to recognize and understand this species and provide a solid basis for its integrated pest management.

## Background

Adapting to grow in relatively cooler, moister, more fertile sandy loams with reduced sunshine, American ginseng *Panax quinquefolium* L. has been introduced to and planted in Liuba County, Shaanxi Province, China, since 1970s; however, it suffers from infestations of pests in the areas the same time [1, 2]. Local divisions of science and technology organized ginseng farmers controlling of those pests, which improved rates of emergence and seedling reservation of the medicinal plant [3]. But, pests have always been difficult to wipe out, previous species continue injuring, and some new pests are quietly occurring, causing more damages and losses of American ginseng. New pests found in recent years include the worm *Enchytraeus buchholzi* Vejdovský, 1878 [4, 5], the root mite *Schwiebea similis* Manson [6–8], a sciarid fly *Bradysia* sp., nematodes, etc. Specimens of the worm collected from Liuba were misidentified in 2014 as *Enchytraeus bulbosus* Nielsen & Christensen [9]. Although the scientific name used previously was incorrect, the project team studied actually the worm on related aspects such as chemical control [10, 11], physiological time [12], field temperature conditions [13], bionomics [14], economic benefits and protective measures [15], feeding amount and generational fertility [16].

The worm *E. buchholzi* has become a new record species for Shaanxi, China and a key pest on *P. quinquefolium* in Liuba County, Shaanxi Province. In order to distinguish this new pest, the authors prepared its whole mounts and paraffin-embedded sections, and microscopically observed, photographed and measured its sizes of forms and structures. These new observations would expand its biological knowledge, help professionals recognize the worm, and provide a basis for its integrated pest management (IPM).

## Results

### Taxonomic Status

Three DNA sequences obtained in the formal experiment, with 676 base pairs each, were compared with those in BOLD Systems [17], which were matched to the species *Enchytraeus buchholzi*, with their similarities ranging from 98.89% to 100%. Thus, the DNA molecular analysis and identification suggested the specimens collected from Liuba be *Enchytraeus buchholzi* at species level. All the following descriptions are given under the new scientific name.

### Body Form

**Fig. 1:**
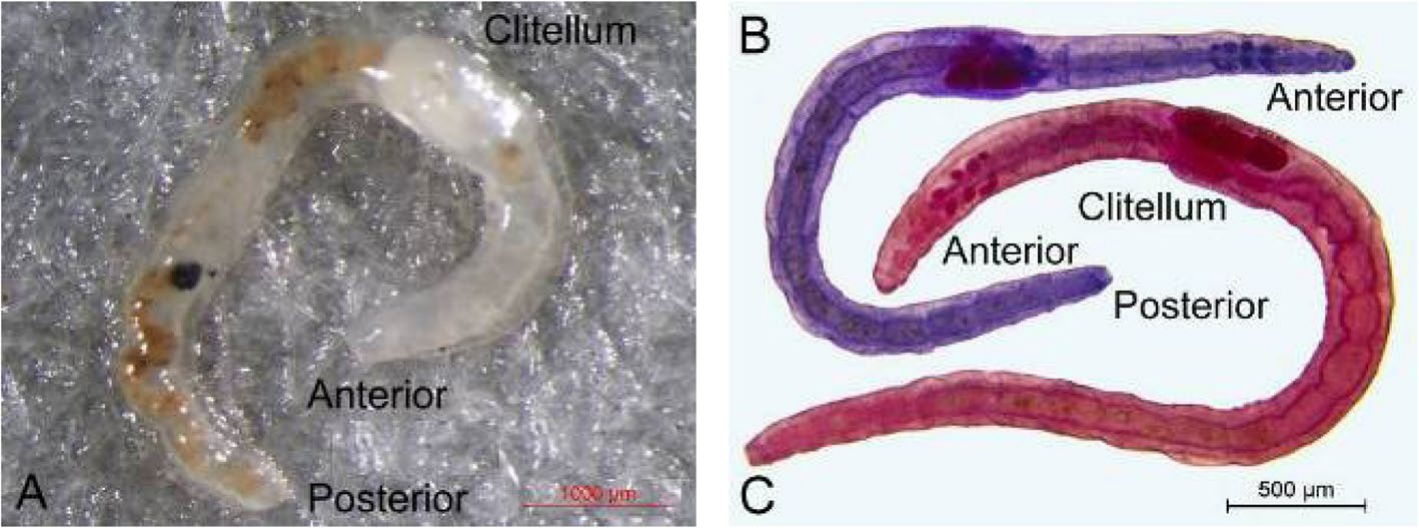
Adults of *Enchytraeus buchholzi* Vejdovský, 1878. **A**. Living specimen, containing yellowish root powders of American ginseng and black impurities in its intestine. **B**. Stained specimen 1 (std-in-HE). **C**. Stained specimen 2 (std-in-Acl)

**Fig. 2:**
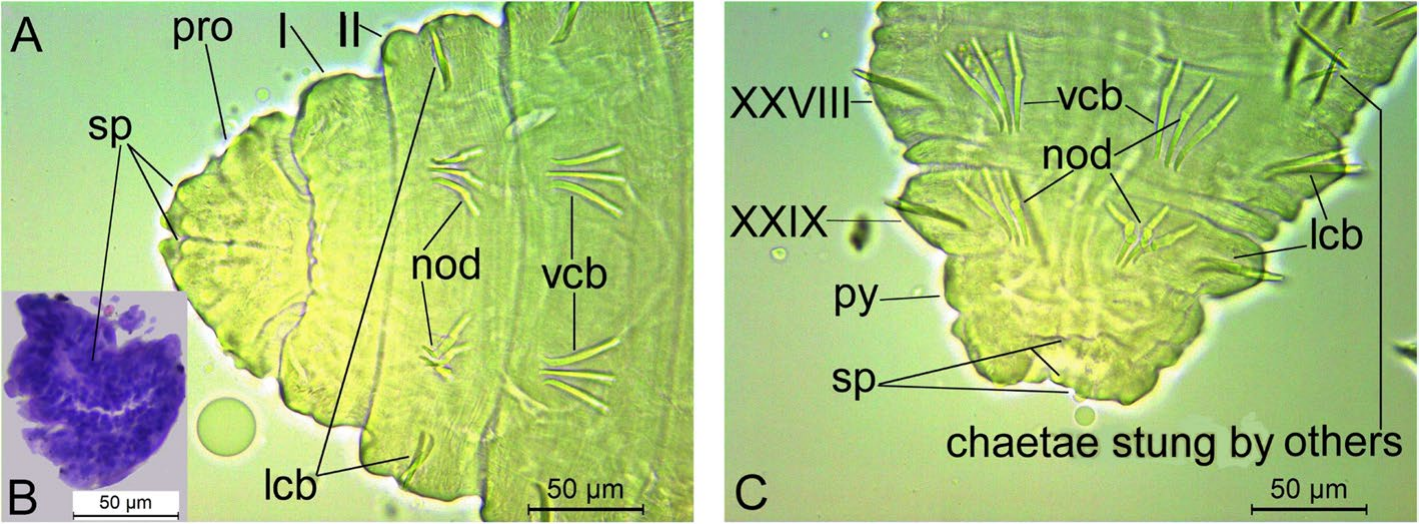
Form of prostomium, segments I – III, XXVIII and XXIX, and pygidium. **A**. Prostomium, segments I (peristomium), II and III (mod-in-Hm), ventral view. **B**. Prostomium, transversal section (std-in-HE), anterior view. **C**. Segments XXVIII and XXIX, and pygidium (mod-in-Hm), ventral view. Labels abbreviated and their meanings (the same as below): *lcb*, lateral chaetal bundle; *nod*, nodule; *pro*, prostomium; *py*, pygidium; *sp*, sensory papillae; *vcb*, ventral chaetal bundle

*Enchytraeus buchholzi* is threadlike and monoecious or hermaphroditic. Living adults are 5 to 8 mm long; when fixed, length ca. 5 mm or slightly longer, width 180 ± 27 μm (sample size, *n* = 86) at segment V, and widest 285 ± 48 μm (*n* = 86) at clitellum. Its body consists of a prostomium, 27 – 30 segments and a pygidium, with the number of segments changing slightly owing to individual variations (Fig. 1). Domelike projections, the sensory papillae, are distributed densely on the epidermis of the prostomium (Fig. 2A, B); there is no chaeta on the epidermis of peristomium (segment I) (Fig. 2A). Each segment from II to XXIX has a pair of lateral chaetal bundles with 2 or 3 chaetae, and a pair of ventral chaetal bundles with 3 chaetae except in XII and XIII (Fig. 11B). The pygidium has no chaeta but many sensory papillae on the epidermis (Fig. 2C; Fig. 10B). Chaetal formula is 2—2,3: 3—3. Chaetae are straight with rarely weakly bent at the distal end, 35—44 μm long, only a few with nodules (Fig. 2A, C; Fig. 11B) [18, 19]. The clitellum covers XII and XIII, which can be distinguished by the two pairs of lateral chaetal bundles arranged longitudinally on each side though there is no intersegmental furrow outside and no septum inside (Fig. 1A; Fig. 11B).

#### Anterior Portion

The prostomium, the clitellum and segments between them constitute the anterior portion of the worm, ca. 1/3 of the full length, where the internal organs are relatively gathered.

**Fig. 3:**
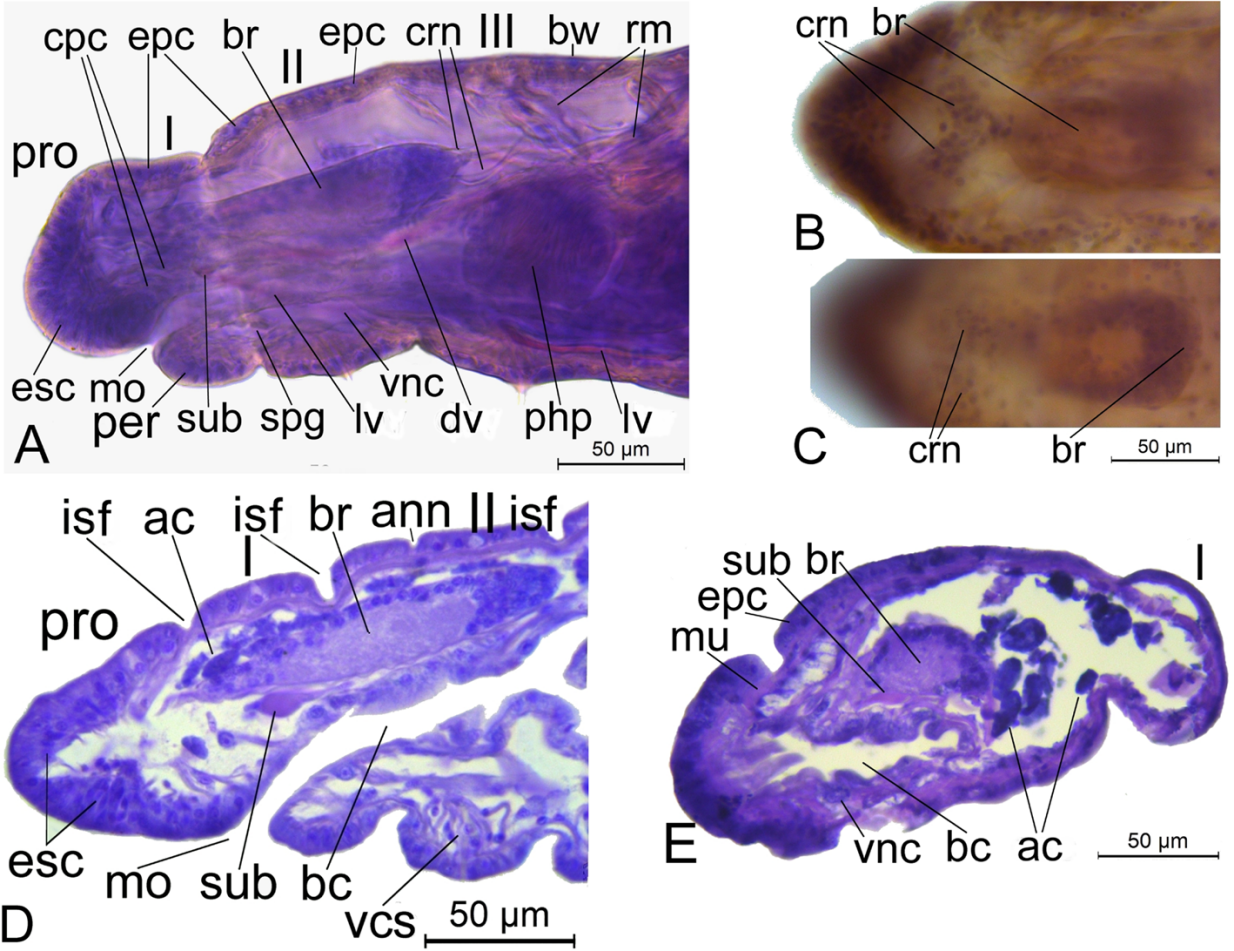
Structure of prostomium, I, II and III (all std-in-HE). **A**. Prostomium, I, II and III, lateral view. **B**. Brain, showing bifurcation in its front part, dorsal view. **C**. Brain, showing round-shape in its posterior margin, dorsal view. **D**. Prostomium, I and II, longitudinal section, lateral view. **E**. Segment I, transversal section, anterior view. *ac*: anucleate coelomic corpuscle; *ann*, annulus; *bc*, buccal cavity; *br*, brain; *bw*, body wall; *cpc*, circumpharyngeal connective; *crn*, cranial nerve; *dv*, dorsal vessel; *epc*, epithelial cell; *esc*, epithelial sensory cell; *isf*, intersegmental furrow; *lv*, lateral vessel; *mo*, mouth; *mu*, muscle; *per*, peristomium; *php*, pharyngeal pad; *pro*, prostomium; *rm*, retractor muscle; *spg*, subpharyngeal ganglion; *sub*, sinus underneath the brain; *vcs*, ventral chaetal sac; *vnc*, ventral nerve cord

**Fig. 4:**
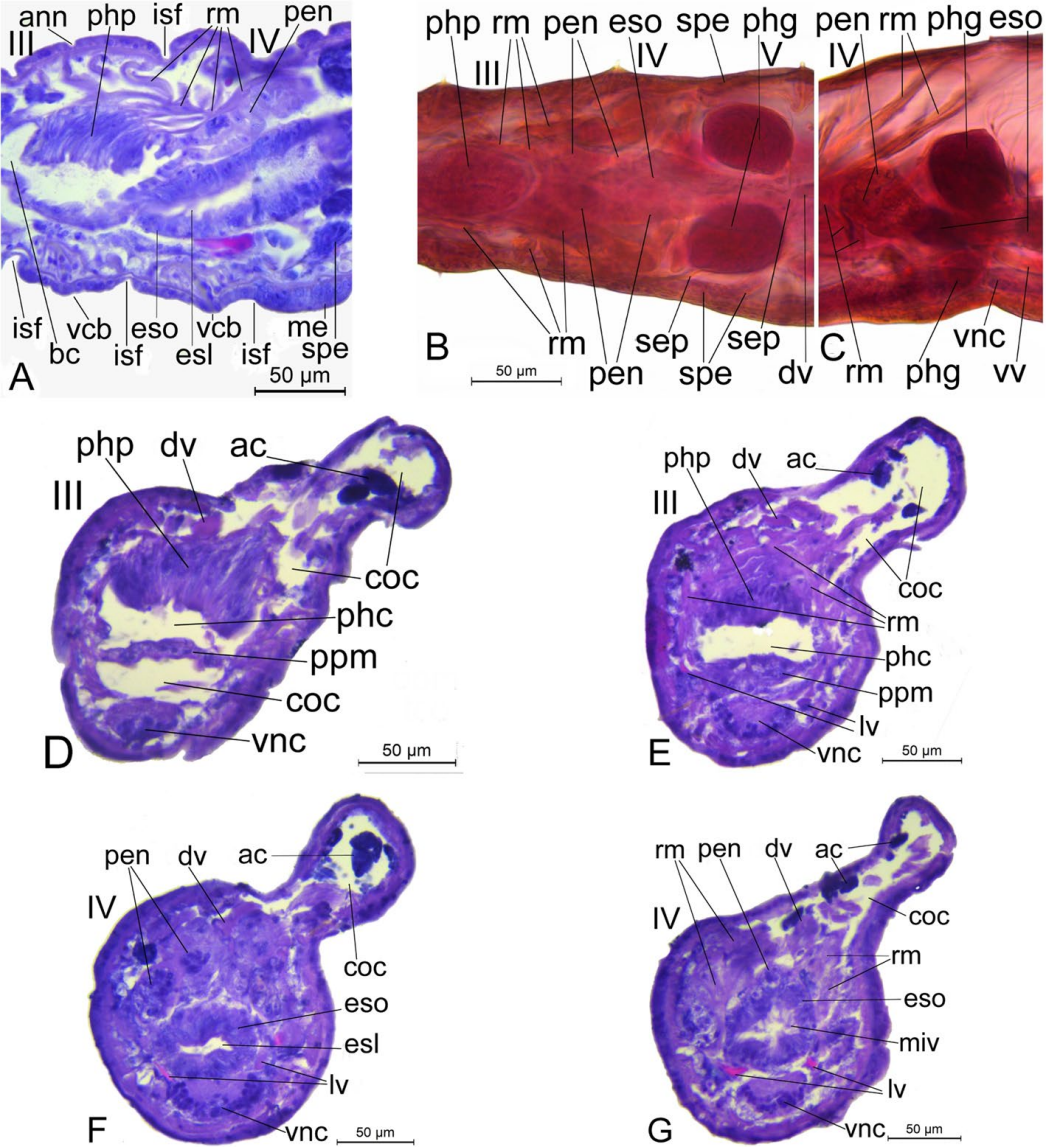
Structure of III and IV, showing pharyngeal pad, retractor muscles, peptonephridium, esophagus, ventral nerve cord, ventral chaetal bundles, vessels, and pharyngeal glands in IV (all std-in-HE except B and C). **A**. III and IV, longitudinal section, lateral view. **B**. III, IV and V, dorsal view (std-in-Acl). **C**. IV, lateral view (std-in-Acl). **D** – **G**. III or IV, transversal section, anterior view. *ac*: anucleate coelomic corpuscle; *ann*, annulus; *bc*, buccal cavity; *coc*, coelomic cavity; *dv*, dorsal vessel; *esl*, esophageal lumen; *eso*, esophagus; *me*, muscular epidermis around the ectal orifice; *isf*, intersegmental furrow; *lv*, lateral vessel; *miv*, microvillus; *pen*, peptonephridium; *phc*, pharyngeal cavity; *phg*, pharyngeal gland; *php*, pharyngeal pad; *ppm*, pharyngeal parietal membrane; *rm*, retractor muscle; *sep*, septum; *spe*, spermatheca; *vcb*, ventral chaetal bundle; *vnc*, ventral nerve cord; *vv*, ventral vessel

**Fig. 5:**
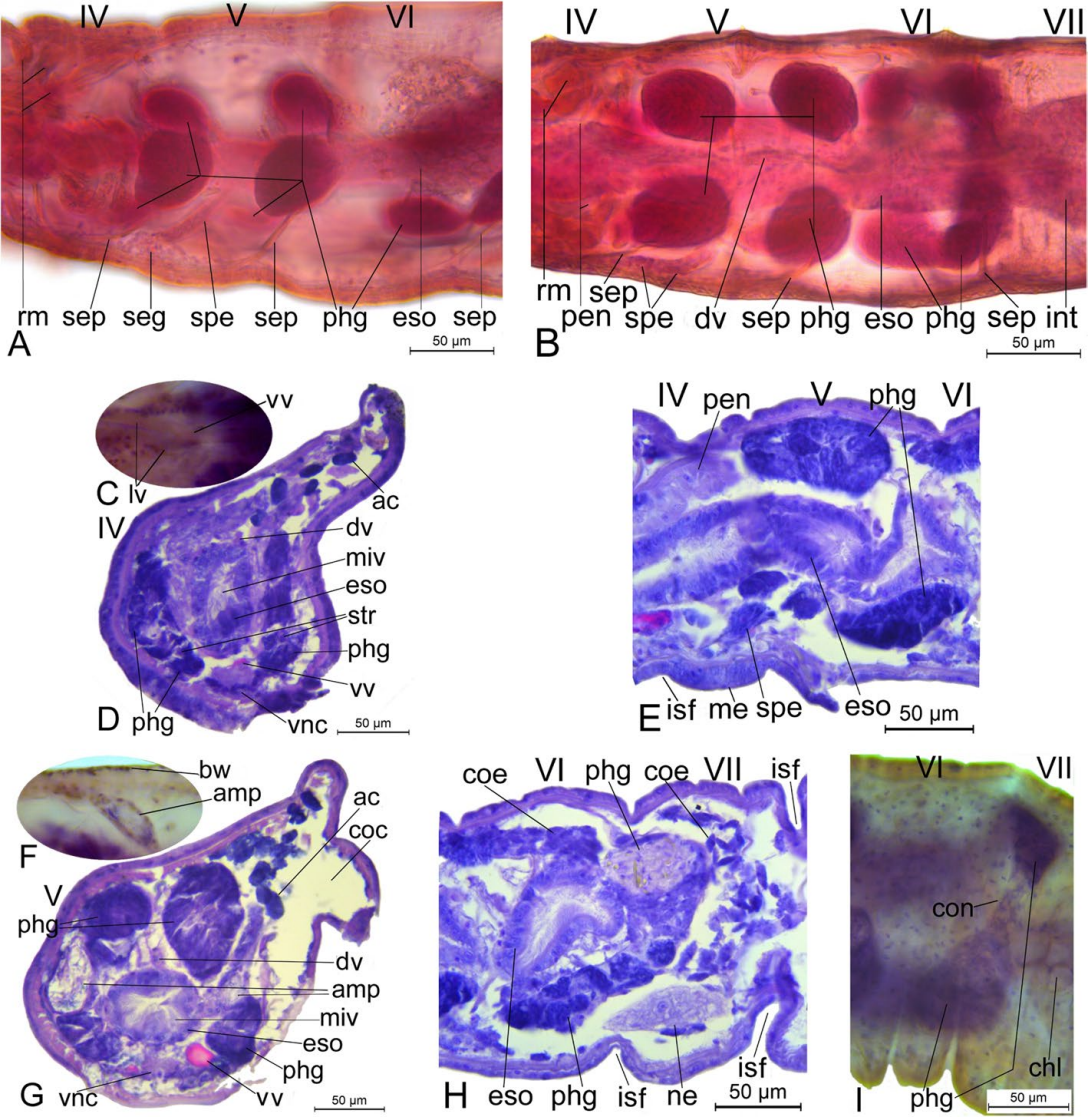
Structure of IV, V, VI and partial VII, showing retractor muscles, pharyngeal glands, spermatheca, esophagus, vessels, and ventral nerve cord (all std-in-HE except **A** and **B**). **A**. IV, V and partial VI (std-in-Acl), lateral view. **B**. IV, V, VI and partial VII (std-in-Acl), dorsal view. **C**. Merge of lateral and ventral vessels in IV, ventral view. **D**. IV, transversal section, anterior view. **E**. partial IV, V and partial VI, longitudinal section, lateral view. **F**. Ampulla of spermatheca, dorsal view. **G**. V, transversal section, anterior view. **H**. VI and VII, longitudinal section, lateral view. **I**. VI and partial VII, showing the connection between dorsal lobes of pharyngeal glands, dorsal view. *ac*, anucleate coelomocyte; *bw*, body wall; *amp*, ampulla of spermatheca; *chl*, chloragogen cell; *coc*, coelomic cavity; *coe*, coelomocyte; *con*, connection between dorsal lobes of pharyngeal glands in VI; *dv*, dorsal vessel; *eso*, esophagus; *me*, muscular epidermis around the ectal orifice; *int*, intestine; *isf*, intersegmental furrow; *lv*, lateral vessel; *miv*, microvillus; *ne*, nephridium; *pen*, peptonephridium; *phg*, pharyngeal gland; *rm*, retractor muscle; *seg*, spermathecal ectal gland; *sep*, septum; *spe*, spermatheca; *str*, strand; *vnc*, ventral nerve cord; *vv*, ventral vessel

Nervous System: There is the brain, long eggplant-shaped in lateral view and inverted pear-shaped in dorsal or ventral view, 96 ± 14 μm long (*n* = 40), 19 ± 3 μm wide at the neck (*n* = 40), 48 ± 7 μm wide (*n* = 20) at the widest part, and 38 ± 6 μm thick (*n* = 20), located in I, II and partial III (Fig. 3A-C). The brain bifurcates in front part (Fig. 3B), and is rounded in posterior margin (Fig. 3C). It extends several cranial nerves, two thicker ones forward to the sensory papillae on the epidermis of the prostomium, two backward to the pharyngeal pad, and also stretches circumpharyngeal connectives downward to the subpharyngeal ganglia in II, linking the ventral nerve cord, 26 ± 9 μm wide and 18 ± 3 μm thick (*n* = 15), till the last segment before the pygidium (Fig. 10). The ventral nerve cord exposes only a little in I (Fig. 3D, E), and is relatively wider and thicker in II, III and IV than that in the rest segments (Fig. 3A; Fig. 4D-G; Fig. 5D, G).

**Fig. 6:**
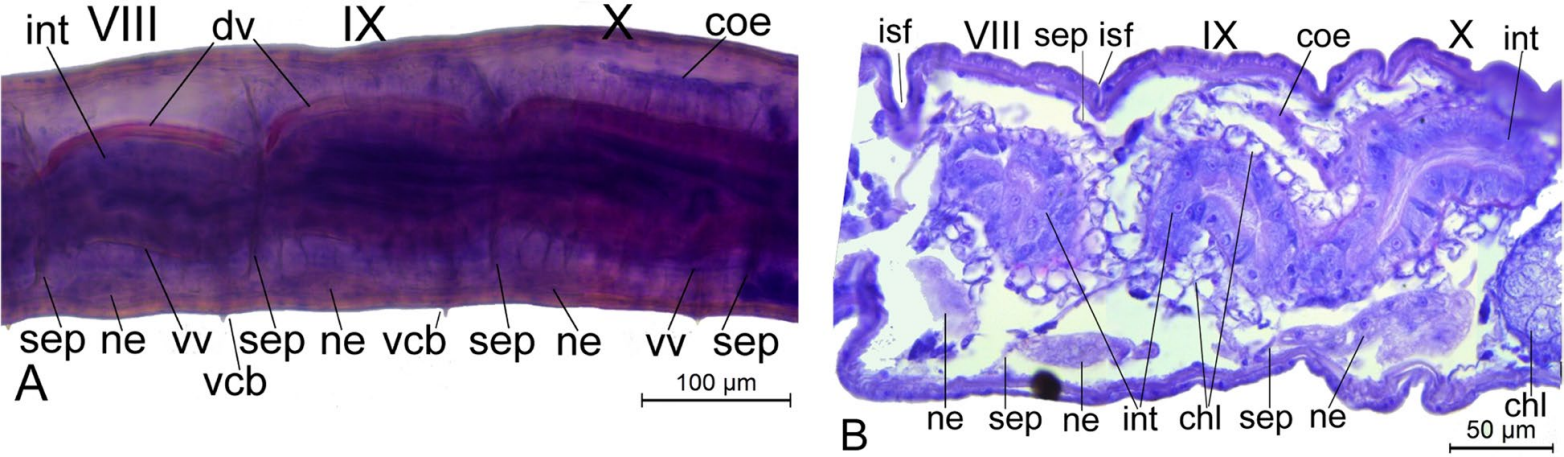
Structure of VIII, IX and X, showing intestine, vessels, chloragogen cells and nephridia (all std-in-HE). **A**. VIII, IX and X, lateral view. **B**. VIII, IX and X, longitudinal section, lateral view. *chl*, chloragogen cell; *coe*: coelomocyte; *dv*, dorsal vessel; *int*, intestine; *isf*, intersegmental furrow; *ne*, nephridium; *sep*, septum; *vcb*, ventral chaetal bundle; *vv*, ventral vessel

**Fig. 7:**
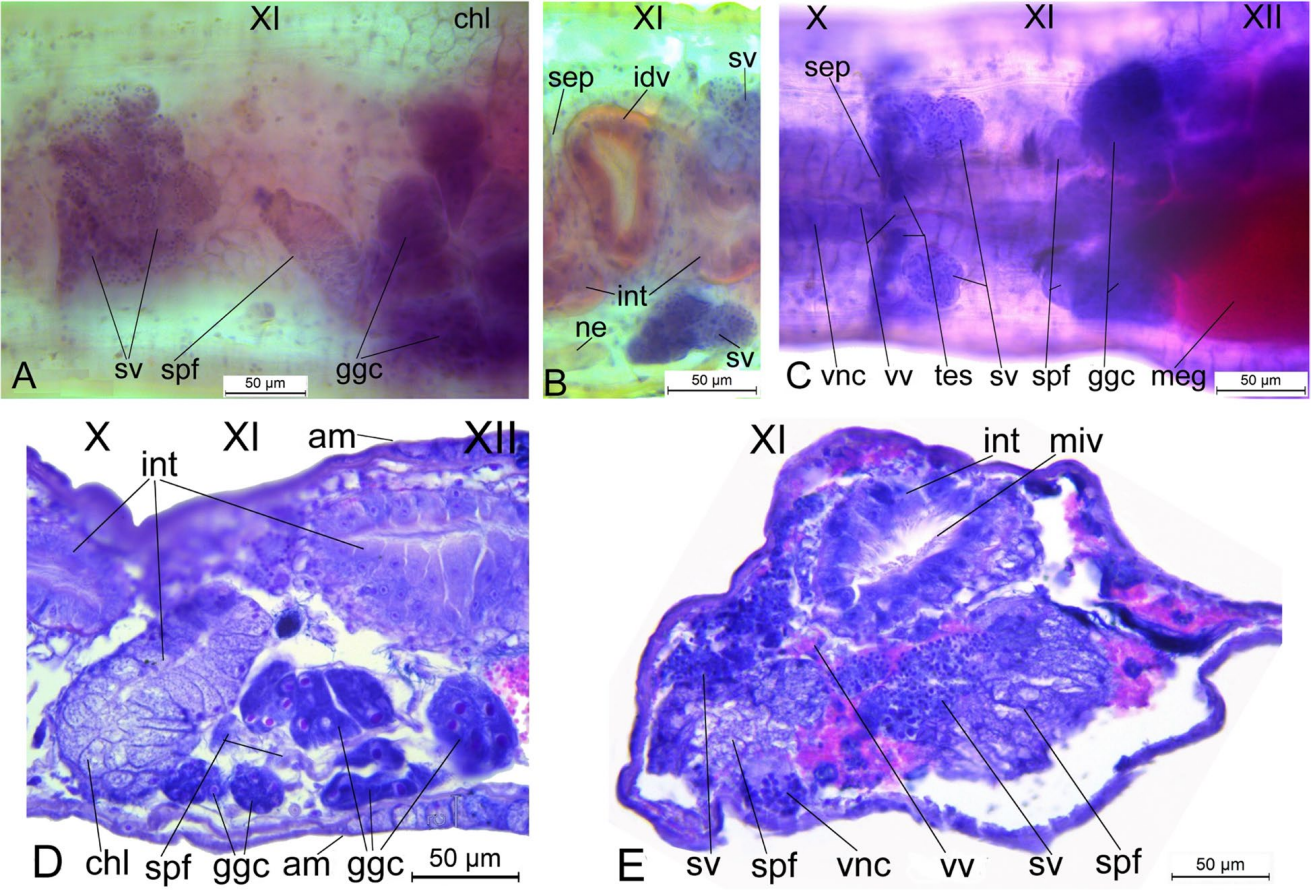
Structure of partial X, XI and partial XII, showing chloragogen cells, intestine, seminal vesicles, testes, sperm funnels, groups of germ cells with developing oocytes, mature eggs, and vessels (all std-in-HE). **A**. XI, lateral view. **B**. Anterior part of XI, showing the inlet of dorsal vessel, dorsal view. **C**. Partial X, XI and partial XII, ventral view. **D**. Partial X, XI and partial XII, longitudinal section, lateral view. **E**. XI, transversal section, anterior view. *am*, anterior margin of XII; *chl*, chloragogen cell; *ggc*, groups of germ cells with developing oocytes; *idv*, inlet of dorsal vessel; *int*, intestine; *meg*, mature egg; *miv*, microvillus; *ne*, nephridium; *sep*, septum; *spf*, sperm funnel; *sv*, seminal vesicle; *tes*, testis; *vnc*, ventral nerve cord; *vv*, ventral vessel

**Fig. 8:**
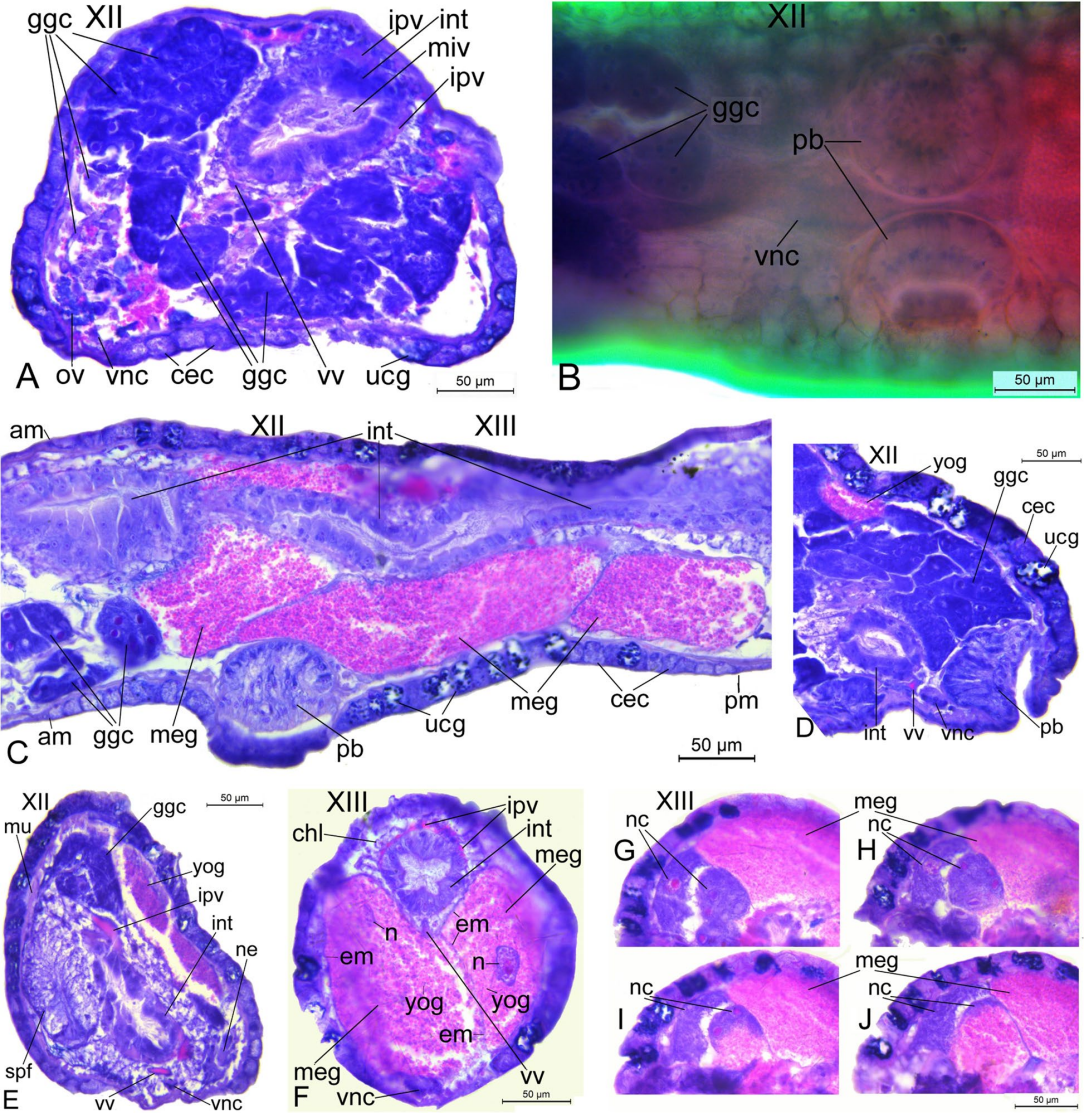
Structure of XII and XIII, the clitellum, largely showing ovary, ventral nerve cord, cover epithelial cells, groups of germ cells with developing oocytes, mature eggs, nurse cells, penial bulbs, unicellular glands, vessels, intestine, muscles, nephridium, and sperm funnels (all std-in-HE). **A**. Near anterior margin of XII, transversal section, anterior view. **B**. Anterior part of XII, ventral view. **C**. XII and XIII, longitudinal section, lateral view. **D**. Penial bulbs and intestine in XII, transversal section, anterior view. **E**. XII, transversal section, anterior view. **F**. XIII, transversal section, anterior view. **G**—**J**. Top-left parts of XIII, transversal series sections, anterior view, showing continuous changes of nurse cells in different layers. *am*, anterior margin of XII; *cec*, cover epithelial cell; *chl*, chloragogen cell; *em*, egg membrane; *ggc*, groups of germ cells with developing oocytes; *int*, intestine; *ipv*, intestinal parietal vessel; *mu*, muscle; *meg*, mature egg; *miv*, microvillus; *ne*, nephridium; *n*, nucleus; *nc*, nurse cell; *ov*, ovary; *pb*, penial bulb; *pm*, posterior margin of XIII; *spf*, sperm funnel; *ucg*, unicellular gland; *vnc*, ventral nerve cord; *vv*, ventral vessel; *yog*, yolk granule

**Fig. 9:**
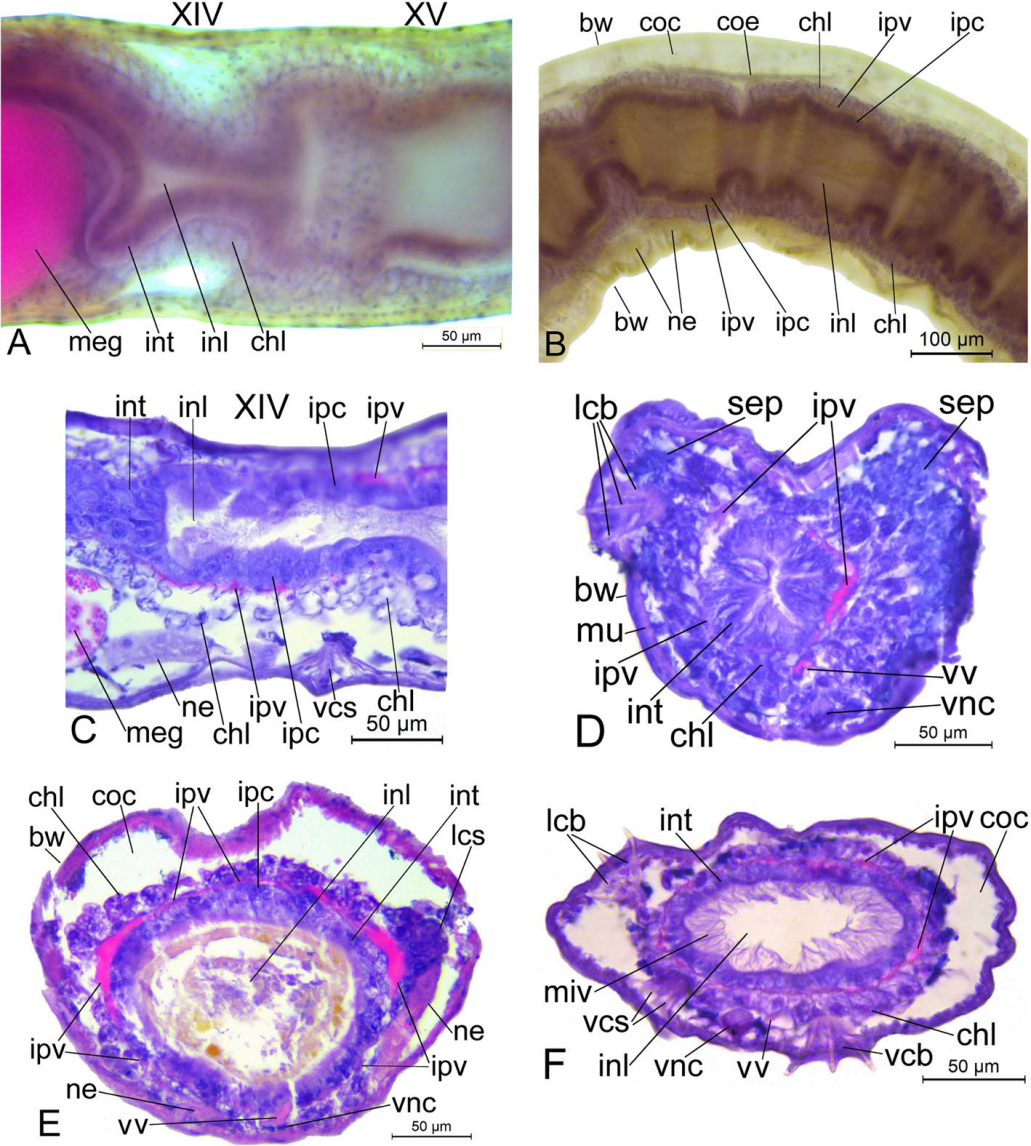
Structure of XIV, XV, and segments thereafter, largely showing chloragogen cells, coelomic cavity, intestine, intestinal parietal vessel and other vessels, lateral and ventral chaetal bundles or sacs, nephridia, septa and ventral nerve cord (all std-in-HE except B). **A**. XIV and XV, lateral view. **B**. Segments after clitellum (mod-in-Hm), lateral view. **C**. XIV, longitudinal section, lateral view. **D**. A segment after clitellum, largely showing septum, transversal section, anterior view. **E** and **F**. A segment after clitellum, largely showing intestinal partial vessels and 3 chaetae in lateral chaetal bundle, transversal sections, anterior view. *bw*, body wall; *chl*, chloragogen cell; *coc*, coelomic cavity; *coe*, coelomocyte; *inl*, intestinal lumen; *int*, intestine; *ipc*, intestinal parietal cell; *ipv*, intestinal parietal vessel; *lcb*, lateral chaetal bundle; *lcs*, lateral chaetal sac; *meg*, mature egg; *miv*, microvillus; *mu*, muscle; *ne*, nephridium; *sep*, septum; *vcb*, ventral chaetal bundle; *vcs*, ventral chaetal sac; *vnc*, ventral nerve cord; *vv*, ventral vessel

**Fig. 10:**
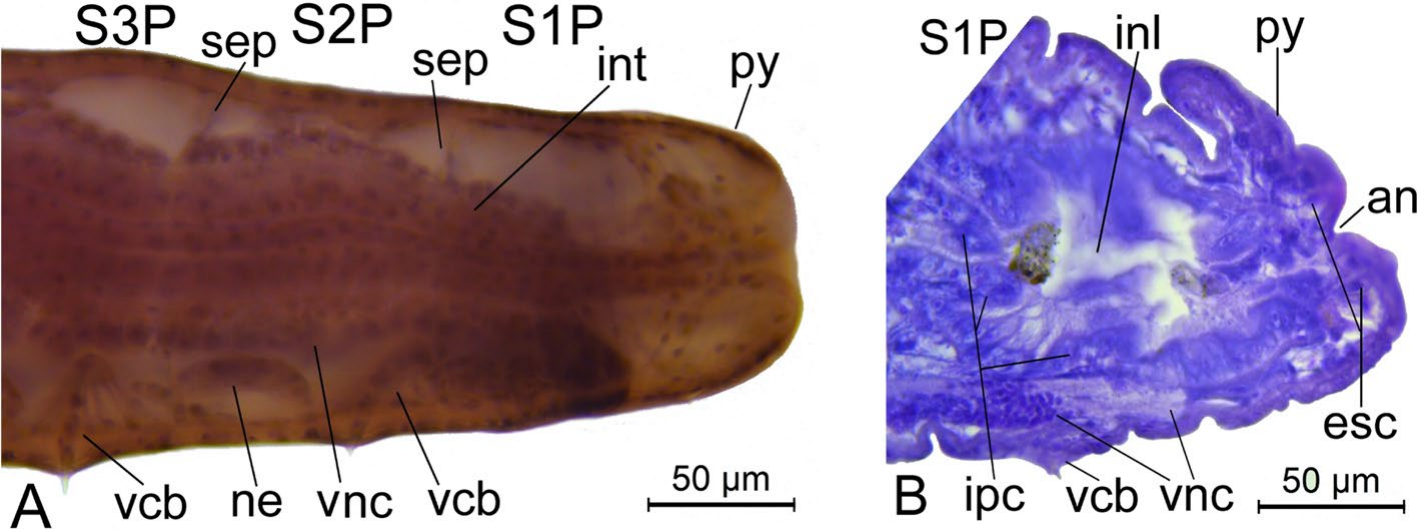
Structure of pygidium and adjacent segments (all std-in-HE). **A**. Lateral view. **B**. Pygidium and segment 1 from the pygidium, longitudinal section, lateral view. *an*, anus; *esc*, epithelial sensory cell; *inl*, intestinal lumen; *int*, intestine; *ipc*, intestinal parietal cell; *ne*, nephridium; *py*, pygidium; *S1P*, segment 1 from the pygidium; *S2P*, segment 2 from the pygidium; *S3P*, segment 3 from the pygidium; *sep*, septum; *vcb*, ventral chaetal bundle; *vnc*, ventral never cord

Digestive System: The mouth opens on the ventral side of the peristomium (Fig. 3A, D), which is followed by the buccal cavity, pharyngeal pad, esophagus, intestine and anus (Figs. 3D, 4, 5, 6, 7, 8, 9 and 10). The dome-like pharyngeal pad, 92 ± 16 μm long (*n* = 32), 77 ± 11 μm wide (*n* = 11) and 40 ± 6 μm thick (*n* = 21), is rich in muscles and has, at the top, a muscular knot and ring (Fig. 4A-E, G). The knot extends many retractor muscles to attach to the body wall on dorsal, lateral and ventral sides (Fig. 4A-C, E, G), and the ring encircles the anterior part of the peptonephridia (esophageal appendages) and esophagus (Fig. 4C, F, G). The peptonephridia, 48.6 μm long and 22.2 μm in diameter at its anterior part (Fig. 4A-C), is attached to the back of the pharyngeal pad; the rest have two branches, coiled and hyaline, 96.4 μm long; the full length is 145 μm and the whole is confined in IV (Fig. 4A-C, F, G). Each segment from IV to VI has a pair of pharyngeal (septal) glands, surrounding esophagus and squeezing the postseptum of the segment to the rear, especially in IV and V (Fig. 5A, B). The pharyngeal glands in IV and V are composed of elliptic dorsal and ventral lobes and thin strands, and the dorsal lobes are larger than the ventral ones (Fig. 5A, B; Table 1). The pharyngeal glands in VI are also made up of dorsal and ventral lobes and thin strands, but sometimes there appears a smaller middle lobe between them (Fig. 5B). The dorsal lobes in IV and V are separate (Fig. 5B), but the ones in VI connected dorsally (Fig. 5B, I). Their sizes are listed in Table 1.

**Table 1:** Sizes of the elliptic lobes of the pharyngeal glands in IV, V and VI, in micron (μm)

Pharyngeal gland in	IV / length × width	V / length × width	VI / length × width
Dorsal lobe (*m* ± *s*)	(87 ± 17) × (60 ± 13)	(79 ± 13) × (60 ± 14)	(72 ± 18) × (39 ± 13)
Ventral lobe (*m* ± *s*)	(64 ± 21) × (39 ± 9)	(86 ± 17) × (34 ± 10)	(92 ± 22) × (39 ± 7)
Sample size (*n*)	45 for DL, 24 for VL	50 for DL, 21 for VL	33 for DL, 35 for VL

*m* mean, *s* standard deviation, DL Dorsal lobe, VL Ventral lobe

Circulatory System: The dorsal vessel, 6.8 ± 0.5 μm in diameter (*n* = 5) in contraction, originating from XI, runs along the dorsal midline of the intestine (Fig. 6A) and esophagus (Fig. 5B; Fig. 4B), forward passes through retractor muscles above the pharyngeal pad (Fig. 4D-G; Fig. 3A), extends into the sinus beneath the brain (Fig. 3A), and then forks laterally and turns downobackward into lateral vessels (Fig. 4E-G), which merge underneath esophagus between ventral lobes of pharyngeal glands in IV (Fig. 5C, D) into the ventral vessel (Fig. 5G; Fig. 6A; Fig. 7C, E), 10.5 ± 3.4 μm in diameter (*n* = 5), and going backward till last segment of the body (Fig. 8A, D-F; Fig. 9B-F). Propelled by cyclic contractions of muscles along the intestinal and body walls, the blood flows forward through dorsal vessel in vivo, causing the vessel and each segment to expand and shrink regularly (Fig. 4D−G; Fig. 5D, G). Besides, there rises a series of intestinal parietal vessels in all the segments between the clitellum and the pygidium (Fig. 8A, F; Fig. 9B-F), which absorb nutritional liquids from intestinal parietal cells and oxygen dissolved in coelomic fluids, and turn them into fresh colorless blood. The blood moves forward in the intestinal parietal vessels with the rhythmic waves of contraction (Fig. 9D) and relaxation (Fig. 9E, F) of circular and longitudinal muscles attaching to the body wall in vivo. When reaching the clitellar segments, the blood pushes the intestine and mature eggs aside, passes through, enters the inlet of dorsal vessel in XI (Fig. 7B), flows onwards to the sinus underneath the brain (Fig. 3A, D, E), and then returns from lateral and ventral vessels in vivo.

Coelomocytes are seen to take place in the coelomic cavity and appear in two forms, nucleate and anucleate. A mass of nucleate coelomocytes, spindle-like, usually 12.9 µm long and 3.2 µm wide, are wrapped in a slender bag, which is hung under dorsal wall of some segments. Anucleate coelomocytes are oval or elliptical, 16.4 ± 2.4 µm long and 8.8 ± 2.1 µm wide (*n* = 63), floating in the coelomic cavity (Fig. 3D, E; Fig. 4D-G; Fig. 5D, G; Fig. 9D, F).

Excretory System: The nephridia are spindle-shaped, 126 ± 28 μm long and 31 ± 5 μm wide (*n* = 16) excluding tubules; there is a pair of them in each of the segments from VII to X, in septa 6/7, 7/8, 8/9 and 9/10, lying on both sides of the ventral nerve cord (Fig. 5H; Fig. 6A, B). In addition, there are similar structures in each of the segments between the clitellum and the pygidium (Fig. 9B, C, E; Fig. 10A).

Reproductive System: Male genital organs: There is a pair of elliptic testes, 14.0 ± 0.9 μm long and 9.7 ± 0.0 μm wide (*n* = 5), lying both sides of the ventral vessel and attaching to the anteseptum of XI closely (Fig. 7C). Nearby is a pair of seminal vesicles in different shape and size, 122 ± 34 μm long (*n* = 81), 102 ± 34 μm wide (*n* = 36) in lateral view and 74 ± 25 μm wide (*n* = 45) in dorsal or ventral view, with many spots on their surfaces (Fig. 7A, C, E). Slightly backward is a pair of sperm funnels, composed of a collar and an ampulla, vase-shaped without base, 105 ± 26 μm long (*n* = 36), 57 ± 21 μm wide (*n* = 24) in lateral view and 69 ± 15 μm wide (*n* = 12) in dorsal or ventral view (Fig. 7A, C-E; Fig. 8E), followed by a sperm duct, 3.2 – 3.5 μm in diameter (*n* = 3), leading to a pair of penial bulbs on the ventral side of XII. More or less on the collar of the sperm funnels are cilia-like spermatozoa, which drift within XI sometimes. Penial bulbs are compact and elliptic, 81 ± 9 μm wide and 79 ± 9 μm high (*n* = 26), with thinner ventral epidermis (Fig. 8B-D). XII and XIII are covered by a single layer of clitellar epithelium containing many granular unicellular glands, 16.1 – 19.4 μm in diameter (Fig. 8A-J). Clitellum is saddle-shaped near the ventral nerve cord (Fig. 8D, E).

Female genital organs: Attaching to both sides of the ventral nerve cord and near anterior margin of XII, there appears a pair of grape-like ovaries in which the immature germ cells, oogonia, are located (Fig. 8A); on the same site the figure also shows that XII is filled with groups of germ cells with developing oocytes that are globular, oval, or in other shape, with only a few cut open but the majority covered with many nurse cells stained blue except their nucleus stained slightly red. These groups of germ cells are aggregated into clusters in different shape, and the sizes of the clusters are: 181 ± 55 μm long and 172 ± 56 μm wide (*n* = 28) in lateral view; 149 ± 45 μm long and 93 ± 24 μm wide (*n* = 46) in dorsal or ventral view (Fig. 8A-D). They are also found in XI (Fig. 7A, C, D), and spirally arranged around two centers in dorsal or ventral view (Fig. 7C). The distance is 90 ± 14 μm (*n* = 5) between the two centers.

Most spaces within the clitellar segments are occupied by mature (ripe) eggs (Fig. 8C, F-J). Fig. 8F shows there are two vitellogenic oocytes or mature eggs at the bottom of the transversal section, each of which contains a lot of yolk granules and a nucleus; especially within the one on the bottom right the nucleus is relatively complete and clear. Fig. 8G-J, top left parts of the four transversal serial sections, illustrate a lot of yolk granules present under the stained-blue nurse cells seen when the cells are peeled off one layer after another. Mature eggs are large and yolk-filled ones, 159 ± 11 μm long and 115 ± 14 μm wide (*n* = 26), inside the clitellum (Fig. 8C, F-J). When there are more mature eggs, they may extend backward to other segments after the clitellum in vivo.

**Fig. 11:**
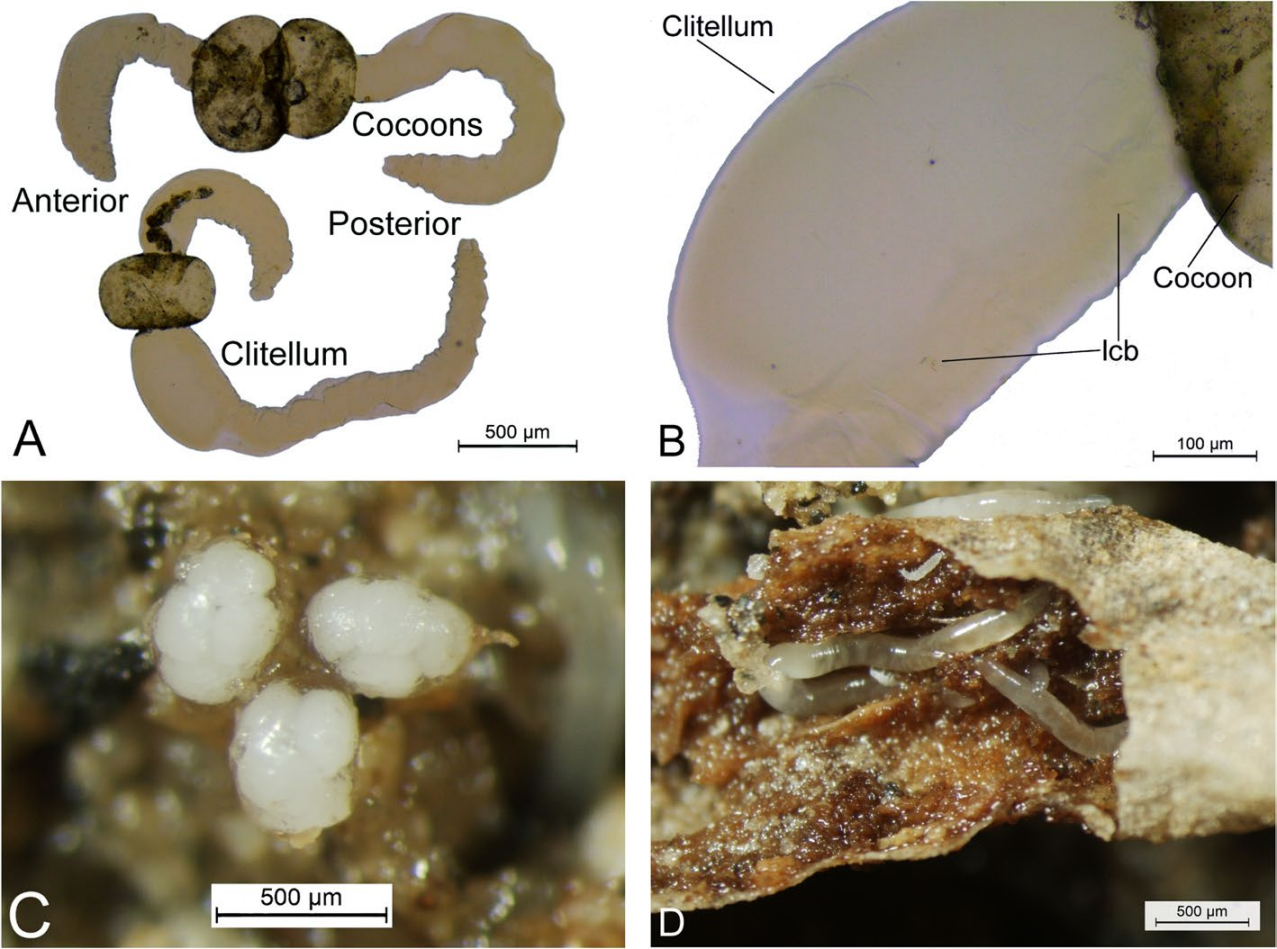
Form of Adults of *Enchytraeus buchholzi* Vejdovský, 1878 with their cocoons. **A**. Adults and their cocoons to be deposited (mod-in-Hm), showing 1 or 2 cocoons are surrounding the body of the worm, lateral view. **B**. Enlargement of the clitellum at the bottom of Fig.11A, showing two lateral chaetal bundles on this side, lateral view. **C**. Three cocoons of the worm, deposited on the wet-sandy surface by adults living inside the wet-sandy dish. **D**. Three adults are injuring a one-year-old small root of American ginseng (the small root is peeled partially to show interior). *lcb*, lateral chaetal bundle

The unicellular glands in the epidermis of the clitellum secrete cocoon wall, which wraps mature eggs inside and forms cocoons (Fig. 11A-C). Cocoons are elliptic, 503 ± 6 μm long and 340 ± 6 μm wide (*n* = 3) if containing ca. 8 mature eggs. Mature eggs become bigger after being laid, 228 ± 17 μm long and 170 ± 15 μm wide (*n* = 12) (Fig. 11C).

There is a pair of spermathecae connecting with their short ectal and long ental ducts, located on both sides of the esophagus and above the ventral lobes of pharyngeal glands in V (Fig. 5A, B, E–G), which open on the ventral body wall, with glandular tissues surrounding their ectal orifice inside (Fig. 5A) and muscular epidermis (cushion) outside (Fig. 5E). The two pieces of muscular epidermis are located along the two longitudinal lines of ventral chaetal bundles, and close to the anteseptum of V (Fig. 4A; Fig. 5E). Their ental ducts stretch diagonally forward to and attach to lateral body wall (Fig. 5B). The ampullae of spermathecae are elliptical or spherical, 42 ± 8 μm long and 32 ± 7 μm wide (*n* = 26), full of cilia-like spermatozoa inside (Fig. 5F, G).

Other Systems: The body wall consists of thin cuticle, epidermal and muscular layers (circular and longitudinal) (Fig. 3A; Fig. 5F; Fig. 9D, E). Both the muscular layers and the coelomic fluids, through the former acting against the latter, form a hydrostatic skeleton, which maintains the shape and toughness of the organism under living conditions. Exchange between oxygen and carbon dioxide is performed through the moist body wall.

### Posterior Portion

Segment XIV, the pygidium and segments between them constitute the posterior portion; all the segments in this portion take on homonomous metamerism except the pygidium. Expressed from outside to inside sequentially, the structure of each layer in these segments is: body wall, ventral nerve cord, nephridia, coelomic cavity, coelomocytes, ventral vessel, chloragogen cells, intestinal parietal vessel, muscular wall of intestine, intestinal parietal cells, and intestinal lumen (Fig. 9B-F). In addition, segments from VII to X in the anterior portion have similar structures (Fig. 5H; Fig. 6A, B), with the intestinal parietal vessel substituted by the dorsal vessel. The segment before the pygidium contains many intestinal parietal cells, and the ventral nerve cord becomes relatively thicker there (Fig. 10A, B).

### Eggs and Their Development

Under living conditions, *E. buchholzi* is pale to milky white (Fig. 1A; Fig. 11D), and reproduces asexually by parthenogenesis; cross fertilization may occur at lower temperatures (12—18 °C). Several mature eggs are wrapped within a cocoon (Fig. 11A-C), which is deposited singly or collectively, and hidden with dusts and debris by parent adults. An adult produces several tens of cocoons in its life time when feeding on American ginseng root powders in a wet-sandy dish indoors. The number of mature eggs is 1—2 per cocoon in early stage, and rises quickly to 3—10 per cocoon with time elapsing, sometimes even up to 15 in a cocoon. In the first 50 days of adults’ reproductive period, the mean number was ever maintained at ca. 8 eggs per cocoon as shown in Fig. 11C, possibly due to the rich nutrition of American ginseng root powders. The outline of mature eggs in a cocoon is clear when the latter is just laid (Fig. 11C); as the embryonic development proceeds, the outline of the eggs inside becomes blurred in vivo. A newly hatched young worm is about 1 mm long; after continuous feeding and direct developing, the worm grows up to an adult when there are about two mature eggs appearing in its clitellum and its color turns from pale to milky white. After that, the adult continues injuring American ginseng severely within roots (Fig. 11D) as well as reproducing more offspring.

## Discussion

### Functional Discrimination and Analysis

Internal organs of the worm are relatively concentrated in its anterior portion, especially in both sections from the prostomium to VI and from XI to XIII. The worm moves freely and quickly inside the host plant and between particles of wet-sandy soils, which is closely related to its developed brain, ventral nerve cord, densely sensory papillae on the prostomium and pygidium, muscular tissues in the body and intestinal walls, and lateral and ventral chaetae.

The pharyngeal pad proper is rich in retractor muscles, and with the help of the muscular knot and ring that extends several muscular bundles out to attach to the body wall, it thus strengthens the effect of grinding food. The anterior part of the peptonephridium opens to basal pharyngeal pad; three pairs of pharyngeal glands connect the pharyngeal pad through tubules or strands [20, 21]. Their functions should be secreting digestive enzymes and enhancing digestive powers by means of extracellular digestion, an important link in its life process.

Seen in I to XI, the dorsal, lateral and ventral vessels were stained red with the HE dyeing way. The ventral and intestinal parietal vessels can also be seen in segments in the posterior portion (Fig. 9B-F), which shows: 1) the ventral vessel extends between the ventral nerve cord and the layer of chloragogen cells, transporting metabolic wastes to the rear; and 2) the intestinal parietal vessels form a network that encircles the digestive tract, absorbing nutritional fluids from intestinal parietal cells, turning them into fresh colorless blood, and carrying it forward. The inlet of dorsal vessel acts as a valve that prevents blood from returning to the rear. The color of the nephridia in the ventral side of VII to X and other segments in posterior portion is pale, showing the excretory function may reduce staining extent in corresponding organs. Nucleate coelomocytes may be the weaver of septa and anucleate coelomocytes may function as lymphatic bodies.

Although the size of the ovaries is small, they reproduce oogonia continuously. Generally, after numerous growing and mitotic divisions, some oogonia develop and enlarge into primary oocytes; undergoing a full meiosis, each oocyte forms three polar bodies and a large haploid ootid; the polar bodies disintegrate and the ootid matures into the egg [22]. The germ-line cysts in *Enchytraeus albidus* ovaries consist of 16 cells; during oogenesis, the fate of interconnected germ cells differentiates and only one cell develops as the future egg, while the other 15 become nurse cells [23]. Recalling the photomicrographs in Figs. 7 and 8, almost all the features of oogenesis in the target worm are similar to those in *E. albidus*. The nurse cells should have produced all the yolk granules, with their chemical properties changing from basophilic to acidophilic, thus stained red. Being bathed in nutrient-rich coelomic fluids in the clitellum, each oocyte and accompanied nurse cells are able to develop rapidly.

### Comparisons with the Type Species and Early Descriptions

The worm *Enchytraeus buchholzi* was first described as a widespread species by Vejdovský in German in 1878 [4]; characters such as body length, segment number, chaetae per bundle, salivary glands (peptonephridia, esophageal appendages), seminal vesicles, sperm funnels, and vas deferens or sperm duct were introduced (Table 2), with other phenomena (intestinal canal covered with glands, segmental organs flowing to outside, ovaries maturing earlier, mature eggs falling into the body cavity, etc.) recorded. A monograph on the class Oligochaeta was compiled by Michaelsen in German in 1900 [24], with the species *E. buchholzi* included and corresponding characters cited especially from the one published by Vejdovský in 1879 [25]. Morphotaxonomic studies and geographical distributions of the worm have been continuing in different parts of the world [26–30], and some early literature is cited to make comparisons with the specimens from Liuba (Table 2).

**Table 2:** Comparisons of the specimens from Liuba with the type species *Enchytraeus buchholzi* and those described by Michaelsen and Nielsen & Christensen [4, 24, 26]

Character^✝^\\Literature	Vejdovský (1878) ‡	Michaelsen (1900) ‡	Nielsen & Christensen (1959)	Specimens from Liuba
Body length (mm)	5 – 8	5 – 10	5 – 10	5 – 8
Segment number	26 – 28	25 – 28	24 – 40	25 – 30
Chaetae per bundle	2 – 3	2 – 4	2,3—2,3: 2,3—2,3	2 – 2,3: 3 – 3
Brain		Longer than wide, cut out at back	Longer than wide; post-end truncate	Longer than wide; post-end round
Dorsal blood vessel		Originating postclitellially	Originating in xii-xiii	Originating in XI
Chloragogen cells		Large, with large, bright oil droplets	With very prominent oil-globules	Covering intestine except in xii-xiii
Coelomocytes, or lymfocytes		Lymphatic body large, flat elliptical	Oval or pear-shaped; with rather few, coarse, refractile granules	With refractile granules, elliptic; 16.4 by 8.8 μm
Salivary glands, or peptonephridia, or esophageal appendages	Multiply sinuous ball	Flat, elongated, widened at the back, with multiple meandering canal	Cylindrical, with much interstitial tissue & a narrow, much coiled canal	Anterior part & two rear branches, coiled & hyaline; 145 μm long
Spermathecae		With bentel-shaped ampulla, and narrow ectal duct with a glandular rim at the bottom	Varying, ectal duct short, with a dense layer of gland cells but without special glands round ectal orifice, rosette-like	Elliptic or spherical, ampulla 42 by 32 μm; ectal duct short, orifice covered with glandular tissues
Nephridia		With slender anteseptale, 2–3 times longer than wide, postseptale oval	Postseptale oval, with short, stout efferent duct arising posteroventrally	Spindle-shaped; one pair in each un-specialized segment, i.e. VII or XV
Seminal vesicles	Varying, thin tubes, or bottle-shaped sacs		Small, attached to x/xi and extending backwards into xi	Different shape; 122 by 102 μm, larger than ½ segment V
Sperm funnels	Calyx-shaped, trans-parent, without glands	Small, 2—3 times as long as thick	Small, pear-shaped or cylindrical with regular collar	Vase-shaped without base, 105 by 57 μm
Vas deferens or sperm duct	Varying, short & thin, long & eyelashing-like	Sparsely twisted	Long, narrow, confined to xii	3.2 – 3.5 μm in diameter
Clitellum		Belt cells arranged in a ring	Over xii-xiii, glandular cells arranged in transverse rows	Over XII-XIII, saddle-like near the ventral nerve cord
Penial bulb, or male copulatory organ		Bursa propulsor large	Compact and rather small	Compact and elliptic, 81 by 79 μm, height less than 1/3 clitellar diameter

^†^ Fifteen taxonomic characters are selected and simplified as above to do comparisons herein owing to each of them is introduced at least by two references, and arranged roughly from front to back; those mentioned only once might be cited directly in the text

^‡^ English phrases translated from original German

Comparisons in Table 2 show the specimens from Liuba are very similar to the type species on many traits, especially on the first three: body length, segment number, and chaetae per bundle. In conjunction with the result of the DNA molecular analysis, the targets are identified into *Enchytraeus buchholzi*, and thus recovered their original identity. There are, of course, some differences among those descriptions, probably owing to distinct habitats and individual variations (Table 2). Based on the traits that seminal vesicles are larger than half diameter of segment V and the ental duct of the spermatheca attaches to lateral body wall (Table 2; Fig. 5B and the text written above), the specimens may be identified into a subspecies, namely *Enchytraeus buchholzi* ssp. *liubaensis.*

The number of chaetae and their shape also contribute significantly to the identification of species in the family Enchytraeidae [4]; according largely to the trait, Vejdovský ever distinguished and named seven new species and redescribed two in the genus *Enchytraeus* [4]. The first two characters, segment number and chaetae per bundle, are the most important traits to distinguish enchytraeid species; the third one, body length, should be less important because of its varying with segment number. Largely relying on the trait, 2—3 chaetae per bundle, with supports of some other traits, several enchytraeids were identified new at species level after 1959; for example, the potworm *Enchytraeus bulbosus* was new to science because its penial bulb is larger than half diameter of the clitellum [9], and the terrestrial species *Enchytraeus luxuriosus* was new because of its segment number around 45 [30].

### Making Slide Specimens

Among the five methods used to make slide specimens, four led to whole mounts and one to paraffin sections, each of which has its own advantage though they are different in fixation, staining and mounting process. The HE dyeing way is recommended since different structures can be colored properly and thus easily recognized; staining in acetocarmine liquid is convenient for fast dyeing, with bright color and good effect. Compared with the slide specimens sectioned, the whole mounts are relatively thicker, and some parts are seen blurred because of beyond the depth of field under higher objective lens and of more tissue layers for light to penetrate. Specimens sectioned can be cut thinner, but some fine structures may be easy to wash away, resulting in image distortion.

## Conclusions

All descriptions and microphotographs of the worm *E. buchholzi*, verified, revised and newly supplemented here, express essential characteristics of the species. Simultaneously, the new results expanded text descriptions, which would be helpful for professionals to recognize this small worm, understand its morphology, structure and physiological function. As forceful pieces of evidence, these microphotographs may support corresponding free-hand drawings published previously and correct something wrong, if any. Concerning IPM practice, the studies would help ginseng farmers aim at the target pest, manage it scientifically, and promote a vigorous development of the American ginseng planting industry.

## Methods

Residual roots of American ginseng damaged by pests were collected from planting areas in Liuba County, Shaanxi Province, China. Living specimens of the worm were picked out from the residual roots, placed within a dish containing wet fine sandy soils and American ginseng root powders (wet-sandy dish), isolated and reared continuously at 21ºC to prepare an indoor stock colony.

A total of five methods were used to make slide specimens, and all were started with picking out active adults from the indoor stock colony: (1) 12 adults were put directly into a drop of Heize medium [31] in the center of a slide, and mounted whole after slightly adjusting their postures (abbreviated to mod-in-Hm later); (2) 16 adults were fixed in Bouin's liquid for 1 d, washed, stained in 10% (v/v) dilution of Ehrlich's hematoxylin liquid for 1 d, rewashed, dehydrated with ethanol series, cleared with xylene, and then mounted whole in Canadian balsam (abbr. to std-in-Hl; indication for slide specimens mounted in Canadian balsam is omitted except those mounted whole in Heize medium, the same below); (3) 86 adults were fixed in Bouin's liquid for 1 d, washed, stained in 10% (v/v) dilution of Ehrlich's hematoxylin liquid for 1 d, counterstained in 0.2% (w/v) eosin within 85% (v/v) ethanol solution for 1 h (HE dyeing way), and then dehydrated, cleared and mounted whole in Canadian balsam as told in (2) (abbr. to std-in-HE); (4) 12 adults were fixed in Bouin's liquid for 1 d, made into paraffin-embedded sections (longitudinal or transversal, 7 μm thick) according to routine steps, stained in the HE dyeing way each for 10 min, and then treated in procedures as told in (2) (abbr. to std-in-HE); and (5) 8 adults were fixed and stained in 1% (v/v) dilution of acetocarmine liquid for 1 d, and then treated in procedures as told in (2) (abbr. to std-in-Acl).

These slide specimens were placed under the optical microscope of the digital microphotography system (DFC 295, Leica Microsystems); external forms and internal structures of the specimens were observed, with their microphotographs taken and sizes calculated later based on scale bars preset. During the same time, many other living adults and egg cocoons were observed in vivo microscopically; some of them were placed under a stereomicroscope (Eclipse E600, Nikon), and their microscopic images were taken with the digital camera (Digital Sight DS-Fi1, Nikon). Segments are marked with Roman numerals.

An experimental study on DNA barcoding of the specimens from Liuba was conducted because their taxonomic status since 2014 was doubtful recently. Based on several preliminary detections, three more living adults were taken from the indoor stock colony, and chopped respectively. Their DNA in mitochondrion was extracted by using MagicMag Micro Genomic DNA Extraction Kit (Sangon Biotech, Shanghai, China). Applying the genomic DNA as a template, the COI gene of the specimen samples was amplified with primers LCO1409 (5'-GGTCAACAAATCATAAAGATATTGG-3'), HCO2198 (5'-TAAACTTCAGGGTGA-CCAAAAAATCA-3') [32], and 2 × Taq PCR Master Mix (DIYIBio, Shanghai, China). All the three PCR samples were sent to Sangon Biotech for determining their DNA sequences of base pairs. Sequence alignment was performed in BOLD Systems [17] to acquire new taxonomic status of the specimens.

All the experiments and studies were conducted and completed in Shaanxi University of Technology in December 2021.

## Data Availability

Some microphotographs generated and analyzed during this study are included in this published article. The DNA sequences of the worm and other related information will be uploaded to BOLDSYSTEMS for professional and public share. The rest are available from the corresponding author on reasonable request.

## Abbreviations

*M*: Mean
*S*: Standard deviation
DL: Dorsal lobe
VL: Ventral lobe
